# Protective Oral Vaccination against *Infectious bursal disease virus* Using the Major Viral Antigenic Protein VP2 Produced in *Pichia pastoris*

**DOI:** 10.1371/journal.pone.0083210

**Published:** 2013-12-20

**Authors:** Omid Taghavian, Holger Spiegel, Rüdiger Hauck, Hafez M. Hafez, Rainer Fischer, Stefan Schillberg

**Affiliations:** 1 Fraunhofer Institute for Molecular Biology and Applied Ecology IME, Aachen, Germany; 2 Institute of Poultry Diseases, Faculty of Veterinary Medicine, Freie Universität Berlin, Berlin, Germany; 3 Institute for Molecular Biotechnology, RWTH Aachen University, Aachen, Germany; 4 Institute for Phytopathology and Applied Zoology, Phytopathology Department, Justus-Liebig University Giessen, Giessen, Germany; University of Delhi, India

## Abstract

*Infectious bursal disease virus* (IBDV) causes economically important immunosuppressive disease in young chickens. The self-assembling capsid protein (VP2) from IBDV strain IR01 was expressed in *Pichia pastoris* resulting in the formation of homomeric, 23-nm infectious bursal disease subviral particles (IBD-SVPs) with a yield of 76 mg/l before and 38 mg/l after purification. Anti-IBDV antibodies were detected in chickens injected with purified IBD-SVPs or fed with either purified IBD-SVPs or inactivated *P. pastoris* cells containing IBD-VP2 (cell-encapsulated). Challenge studies using the heterologous classical IBDV strain (MB3) showed that intramuscular vaccination with 20 µg purified IBD-SVPs conferred full protection, achieved complete virus clearance and prevented bursal damage and atrophy, compared with only 40% protection, 0–10% virus clearance accompanied by severe atrophy and substantial bursal damage in mock-vaccinated and challenge controls. The commercial IBDV vaccine also conferred full protection and achieved complete virus clearance, albeit with partial bursal atrophy. Oral administration of 500 µg purified IBD-SVPs with and without adjuvant conferred 100% protection but achieved only 60% virus clearance with adjuvant and none without it. Moderate bursal damage was observed in both cases but the inclusion of adjuvant resulted in bursal atrophy similar to that observed with live-attenuated vaccine and parenteral administration of 20 µg purified IBD-SVPs. The oral administration of 250 mg *P. pastoris* cells containing IBD-VP2 resulted in 100% protection with adjuvant and 60% without, accompanied by moderate bursal damage and atrophy in both groups, whereas 25 mg *P. pastoris* cells containing IBD-VP2 resulted in 90–100% protection with moderate bursal lesions and severe atrophy. Finally, the oral delivery of 50 µg purified IBD-SVPs achieved 40–60% protection with severe bursal lesions and atrophy. Both oral and parenteral administration of yeast-derived IBD-VP2 can therefore induce a specific and protective immune response against IBDV without affecting the growth rate of chickens.

## Introduction

*Infectious bursal disease virus* (IBDV) serotype I is an immunosuppressive virus (genus *Avibirnavirus*, family *Birnaviridae*) that causes significant morbidity and mortality in young chickens. The virus is stable in the presence of disinfectants [1] and is transmitted via the oral-fecal route [2], [3]. When susceptible chickens are infected, IBDV replicates in gut-associated macrophages and lymphoid cells, allowing it to reach the bursa of Fabricius (BF). The virus predominantly targets maturing B lymphocytes in the BF [4] via *α*4*β*1 integrin [5]. IBDV induces apoptosis in the peripheral lymphocytes [6] and causes severe immunosuppression and often death in chickens that are 3–6 weeks old, when the BF is in its critical development stage [7].

Infected chickens become susceptible to other diseases and their response to vaccination declines. Younger chickens are passively protected by maternal antibodies transmitted via the egg yolk [8] whereas older ones can produce antibodies against the virus and only rarely develop clinical signs of the disease. The clinical signs include distress, depression, diarrhea, anorexia, ruffled feathers, trembling and dehydration, usually appearing 2 days after infection and declining by day 4 due to the rapid recovery of survivors [9].

There is no specific treatment for IBD and currently the disease is controlled by administering attenuated or inactivated IBDV as a vaccine [2]. Attenuated vaccines are usually administered orally in drinking water, whereas inactivated vaccines are administrated by intramuscular injection.

IBDV has two serotypes but only serotype 1 is pathogenic in chickens. Within this serotype, classical and antigenically-distinct variant strains can be distinguished. The “very virulent” (vv) strains that have been circulating since the late 1980s in Europe, Africa, and Asia are antigenically similar to the classical strains [3]. Virus-neutralization tests can also distinguish several further subtypes [10]. The prevalence of vv strains probably reflects the selection pressure caused by vaccination [11], hence conventional vaccines are now unable to provide full protection [12]. Most live vaccines are based on classical virulent strains and are classified as “mild” vaccines with low protective efficiency against vv strains. Intermediate and intermediate plus or “hot” vaccines confer better protection, but they do not provide complete protection and may also induce moderate to severe clinical signs and immunosuppression [13], [14]. Therefore, only mild and intermediate viruses are used as attenuated virus vaccines [15]. Inactivated vaccines are expensive to produce and deliver, and they only provide weak protection [16]. However, they are used to induce high levels of antibodies in breeder hens, so that chickens are protected by maternal antibodies for a considerable time. In this case, it is crucial to identify the optimal time point for vaccination to boost protective immune responses without interfering with the maternal antibodies [17]. The development of novel and efficacious vaccines against vv strains is therefore essential. Genetically engineered viral vectors have been used successfully to induce T-cell as well as B-cell immunity without interfering with maternal antibodies [1]. Also, in contrast to conventional vaccines, recombinant subunit vaccines do not by design include genetic material and can therefore induce a protective immune response but are unable to revert to virulence.

IBDV has a non-enveloped icosahedral capsid with a *T* = 13 surface lattice and a linear double stranded RNA genome in two segments, named A and B. Segment B encodes an RNA-dependent RNA polymerase, and segment A contains two partly overlapping open reading frames (ORFs) encoding the non-structural protein VP5 (which facilitates virus dissemination [18]) and a larger polyprotein that is autocatalytically cleaved into the structural proteins VP2 and VP3, and the serine protease VP4 [9], [19], [20]. VP2 is the major IBDV antigen, which includes a conformational epitope that can induce the production of virus-neutralizing antibodies [21]. VP2 is also responsible for antigenic variation, virulence and tissue culture adaptation [22]. When VP2 is produced in heterologous cells, the recombinant protein can aggregate to form symmetrical, multimeric subviral particles (SVPs) with enhanced immunogenicity.

VP2 has been expressed in several heterologous systems with different degrees of success. Bacterial systems such as *Escherichia coli* produce non-immunogenic SVPs [23], [24]. However, yeasts such as *Saccharomyces cerevisiae* [23] and *Pichia pastoris* [25], baculovirus-infected insect cell lines such as SF9 [26] and High-Five [27], and baculovirus-infected insects such as cabbage looper larvae (*Trichoplusia ni*) [28], can produce SVPs that confer protection following parenteral administration, albeit after extensive purification. In contrast, plants such as *Arabidopsis thaliana* [29] and rice [16] can produce recombinant VP2 suitable for direct oral delivery without purification, although this does not achieve complete protection probably because of the inefficient assembly of IBD-SVPs in plant cells.

Oral vaccine delivery is simple and inexpensive, but orally-delivered subunit vaccines tend to have limited and short-lived immunogenicity that must be addressed by boost regimes and/or the co-administration of adjuvants. We previously described the production of IBD-SVPs in *P. pastoris* [30]. Here we report the oral delivery of yeast-derived recombinant IBD-VP2 from the vv strain IR01 [31] to young chickens. The vaccine was applied using boost regimes comprising either a cell-encapsulated vaccine (inactivated freeze-dried *P. pastoris* cells containing IBD-VP2) or purified IBD-SVPs alone or in combination with an oral adjuvant mixture comprising CpG oligonucleotides (CpG ODNs) and NaF [32]. We found that these candidate vaccines conferred partial or full protection against IBD when young chickens were challenged with IBDV.

## Materials and Methods

### Cloning and transformation

**Figure 1:**
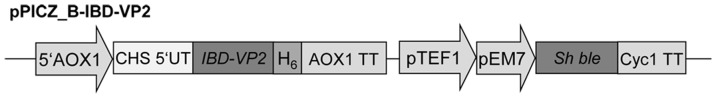
*Pichia pastoris* expression cassette in pPICZ_B (Invitrogen). Abbreviations: 5′AOX1 and AOX1 TT, methanol-inducible alcohol oxidase 1 gene promoter and terminator, respectively; CHS 5′-UT, untranslated region of the *Petroselinum hortense* chalcone synthase gene; *IBD-VP2*, cDNA of *Infectious bursal disease virus* protein 2, corresponding to the first 441 amino acids; H_6_, His-6 tag for detection and purification; pTEF1, transcription elongation factor 1 gene promoter from *S. cerevisiae* that drives expression of the *Sh ble* gene in *P. pastoris* conferring zeocin resistance; pEM7, constitutive synthetic prokaryotic promoter that drives expression of the *Sh ble* gene in *E. coli*; *Sh ble*, *Streptoalloteichus hindustanus* bleomycin resistance gene; Cyc1 TT, *CYC1* transcription termination region (GenBank accession number M34014), the 3′ end of the *S. cerevisiae CYC1* gene that allows efficient 3′ mRNA processing of the *Sh ble* gene for increased stability.

The *IBDV-VP2* cDNA from strain IR01 (GenBank accession number AY704912 [31]) was used as a template and the sequence corresponding to the mature IBD-VP2 was amplified using a two-step PCR procedure. In the first step, an overhang was introduced onto the 5′-end of the sequence using forward primer 5′-TTT ATA AAA AAA AAA AAA AC-3′ and a His_6_-tag was introduced onto the 3′-end using reverse primer 5′-GCT CTA GAT TTA GTG ATG GTG ATG GTG ATG TGC TCC TGC AAT CTT CAG-3′. In the second step, the *Petroselinum hortense* chalcone synthase 5′ untranslated region was introduced upstream of the *IBD-VP2* cDNA using an overlapping complementary primer (5′-CGC GAA TTC ACA ACA CAA ATC AGA TTT ATA GAG AGA TTT ATA AAA AAA AAA AAA AC-3′) and the same reverse primer. The product was transferred to the expression vector pPICZ_B (Invitrogen, Karlsruhe, Germany) using the *Eco*RI and *Xba*I restriction sites (underlined) placing it under the control of the methanol-inducible alcohol oxidase (*AOX1*) promoter (Figure 1). The recombinant vector was introduced into *P. pastoris* strain X-33 (Invitrogen) as previously described [33] to yield the recombinant strain Pichia IBD-VP2.

### IBD-VP2 expression, extraction and purification

Recombinant yeast cells were cultured in YPD medium (1% (w/v) yeast extract, 2% (w/v) peptone and 2% (w/v) dextrose) as recommended (EasySelect™ Pichia Expression Kit, Invitrogen). IBD-VP2 expression was induced by resuspending the cells to OD_600nm_ = 1.0 in BMMY medium (100 mM sodium phosphate, pH 6.0, 1% (w/v) yeast extract, 2% (w/v) peptone, 1.34% (w/v) yeast nitrogen base, 0.4 µg/ml biotin) containing 0.5% (v/v) methanol. The most productive colony was identified by immunoblotting, and was cultured in 500 ml BMMY medium for 4 days as recommended (Invitrogen). Methanol was added to a final concentration of 0.5% (v/v) on the second day and increased to 1% (v/v) on the third and fourth days. The cells were then harvested by centrifugation at 3,000×g for 5 min at room temperature, resuspended in breaking buffer (100 mM sodium acetate, pH 4.0, 1 mM PMSF, 1 mM EDTA, 5% (v/v) glycerol) and disrupted by five passes in a microfluidizer (Newton, MA, USA). The supernatant was collected after centrifugation at 13,000×g for 30 min at room temperature, IBD-VP2 was precipitated using 50% ammonium sulfate and resuspended in 5 ml phosphate-buffered saline (PBS). The purified sample was polished and simultaneously characterized by size exclusion chromatography (SEC) on a Hiprep 26/60 Sephacryl S400 HR column (GE HealthCare, Freiburg, Germany). The IBD-SVP elution fractions were concentrated using a Vivaspin 20 spin column with a 300-kDa cut-off membrane (Sartorius-Stedim, Göttingen, Germany). The purity of the IBD-SVPs was determined by the densitometric analysis of polyacrylamide gels stained with Coomassie Brilliant Blue, using AIDA image analysis software. The protein content was determined using the BCA assay kit (Thermo Scientific, Dreieich, Germany).

### SDS-PAGE and immunoblotting

The protein samples were separated by SDS-PAGE (12% (w/v) polyacrylamide), transferred to a nitrocellulose membrane and blocked in 5% (w/v) skimmed milk in PBS containing 0.05% (v/v) Tween 20 (PBST). Recombinant IBD-VP2 was detected with a rabbit anti-VP2 [27] primary antibody (diluted 1∶10,000) kindly provided by Prof. Wang (National Chung Hsing University, Taichung, Taiwan), and an alkaline phosphatase-conjugated goat anti-rabbit secondary antibody (Dianova, Hamburg, Germany) diluted to 0.2 µg/ml. Each reaction was carried out for 1 h at room temperature with gentle agitation. After three 5-min washes in PBST, the signal was detected with NBT/BCIP (Biorad, München, Germany).

### Transmission electron microscopy

IBD-SVPs images were acquired by applying 30-µl aliquot samples onto discharged 400 mesh carbon-coated nickel grids (Plano, Wetzlar, Germany) for 15 min at room temperature. Excess particles were removed by rinsing with PBS. The grids were negatively stained with 2% (w/v) aqueous uranyl acetate and observed under a 400T electron microscope (Philips, Eindhoven, the Netherlands) operated at 60 kV accelerating voltage. Digital images were captured with an Olympus camera (MORADA) and processed using iTEM software (Münster, Germany).

### Animals

Specific pathogen free (SPF) eggs from white Leghorn chickens were purchased from Lohmann Tierzucht (Cuxhaven, Germany). Hatched chickens were kept under SPF conditions in isolators under positive filtered air pressure and were provided with free access to standard food and drinking water. The bodyweight (BW) gain and health of the chickens were monitored before infection twice daily and after infection six times daily throughout the experiment.

### Immunization and sampling

Ethics statement: The animal experiments were officially approved by the Landesamt für Gesundheit und Soziales, reference number G 289/10. All animals received humane care in accordance with the requirements of the German Tierschutzgesetz and the European Commission guidelines on the accommodation and care of animals used for experimental and other scientific purposes. Following infection, birds were humanely euthanized if they were not able to reach feeders and drinkers, and were counted as disease mortality. The survivors were also euthanized one week after infection. No analgesics or anesthetics were used before euthanization. Birds were stunned by a blow to the head and killed by dislocation of the neck or by cutting the vena jugularis.

Sixty-five 14-day-old chickens were marked individually and randomly divided into 13 experimental groups of five individuals, and were maintained in separate isolators as follows: isolator 1 – groups 1, 2, 3 and 4; isolator 2 – groups 5, 6, 7 and 8; isolator 3 – groups 9, 10 and 11; isolator 4 – group 12; isolator 5 – group 13. The chickens were immunized four times at weekly intervals as shown in Table 1. For oral immunization, the freeze-dried inactivated yeast cells expressing the IBD-VP2 protein, and the purified IBD-SVPs, were administered as shown in Table 1. Prior to immunization, the freshly harvested cell suspensions were inactivated by applying two thermal cycles at 70°C and 15°C, each for 5 min. The suspensions were supplemented with 50 µg CpG oligodeoxynucleotides plus NaF (100 mg per kg body weight at the time of vaccination) as an oral adjuvant. The freeze-dried cell-encapsulated vaccine was applied in 250-mg and 25-mg doses with and without adjuvant mixture in 1 ml drinking water. Purified IBD-SVPs were applied in 500-µg and 50-µg doses with and without adjuvant in 0.5 ml PBS. As a control, 250 mg of freeze-dried inactivated wild-type *P. pastoris* cells was applied to group 9.

**Table 1:** Experimental design for chicken immunization.

				Age (days) at vaccination				Age (days) at immunoassay						
Experimental groups	Administration route^1^	Vaccine composition	Number of chickens	1st	2nd	3rd	4th	1st	2nd	3rd	4th	5th	6th^2^	Days of challenge with live virus
1	O	250 mg of Pichia^3^ containing IBD-VP2	5	14	21	28	35	14	20	27	34	42	49	42
2	O	250 mg of Pichia containing IBD-VP2 + oral adjuvant^4^	5	14	21	28	35	14	20	27	34	42	49	42
3	O	25 mg of Pichia containing IBD-VP2	5	14	21	28	35	14	20	27	34	42	49	42
4	O	25 mg of Pichia containing IBD-VP2 + oral adjuvant	5	14	21	28	35	14	20	27	34	42	49	42
5	O	50 µg of purified IBD-SVPs	5	14	21	28	35	14	20	27	34	42	49	42
6	O	50 µg of purified IBD-SVPs + oral adjuvant	5	14	21	28	35	14	20	27	34	42	49	42
7	O	500 µg of purified IBD-SVPs	5	14	21	28	35	14	20	27	34	42	49	42
8	O	500 µg of purified IBD-SVPs + oral adjuvant	5	14	21	28	35	14	20	27	34	42	49	42
9	O	250 mg of Pichia wild-type	5	14	21	28	35	14	20	27	34	42	49	42
10	P	20 µg of purified IBD-SVPs + Adjuvant 100	5	14	21	28	35	14	20	27	34	42	49	42
11	X	Challenge control	5	-	-	-	-	14	20	27	34	42	49	42
12	O	AviPro Gumboro vac (oral control^5^)	5	14	21	28	35	14	20	27	34	42	49	42
13	X	Vaccine control	5	-	-	-	-	14	20	27	34	42	49	-

1 Route of vaccine administration was shown as O: oral (via oral gavage), P: parenteral (intramuscularly) and X: no vaccination.

2 Chickens survived virus challenge were sacrificed at day 49, bursa was extracted and analyzed for the presence of virus and histological bursa lesion assessment.

3 Pichia: freeze-dried and inactivated intact *P. pastoris* cells. Resuspended in 1 ml drinking water.

4 Oral adjuvant is a mixture of NaF salt (100 mg/kg chicken weight) and 50 µg CpG oligodeoxynucleotide (CpG ODN).

5 The administered dosage was according to the manufacture's instructions (Lohmann Animal Health, Cuxhaven, Germany).

Oral immunization was achieved by oral gavage into the crop. The attenuated virus preparation AviPro Gumboro vac (Lohmann Animal Health GmbH & Co. KG, Cuxhaven) was also applied orally as a positive control, according to the manufacturer's instructions.

Parenteral immunization was achieved by the intramuscular injection of 20 µg purified IBD-SVPs combined with 20 µl Adjuvant 100 (Gerbu, Wieblingen) in a total volume of 100 µl. Two groups of chickens were not vaccinated and were used as negative controls in the viral challenge (group 11) and vaccine (group 13) experiments, respectively.

### Serum antibody responses in immunized chickens

Blood samples were collected from the wing vein of immunized chickens at different time points (Table 1) and the IBD-VP2-specific IgM and IgY antibody responses in the sera were measured by indirect ELISA. The purified yeast-derived IBD-SVPs were coated onto 96-well microtiter plates (10 µg/ml in PBS) overnight at 4°C. The plates were blocked with 2.5% (w/v) skimmed milk in 1× PBS containing 0.05% (v/v) Tween 20 (PBST) for 1 h at room temperature. After three washes with PBST, the plates were incubated with 100 µl serum samples (diluted 1∶50 in PBS) for 2 h at room temperature. After three further washes, IgY and IgM were detected with 2 µg/ml horseradish peroxidase-conjugated rabbit anti-chicken IgY (Gentaur, Aachen, Germany) or 5 µg/ml horseradish peroxidase-conjugated goat anti-chicken IgM (Gentaur). Bound secondary antibodies were detected using the ABTS substrate and quantified on a microplate reader at 405 nm. A response was considered positive if the mean absorbance was more than twice that of the pre-immune sera plus two standard deviations (>0.2).

The anti-IBD titer was also determined using a commercial antibody ELISA test kit (IDEXX IBD-XR Ab test, Westbrook, ME, USA). This kit is often used in chicken farms to evaluate immunity against IBDV by testing the serum antibody titer against the virus. The method is an indirect ELISA and titers above 396 are considered positive.

### Challenge experiments and post-challenge studies

Chickens were challenged with the classical IBDV strain MB3 from a recent outbreak in Germany. This strain does not grow well in embryonated SPF eggs and is not cell culture adapted, so birds were infected via the ocular route with 0.1 ml homogenized bursa of Fabricious (BF) from naturally infected chickens, passed through a 0.2-µm filter. The samples were kindly provided by Dr. Hermann Block [17]. All chickens except those in group 13 were challenged with the virus when they were 42 days old. Clinical signs and mortality were recorded for one week after the challenge. The birds were then killed and the BF isolated to determine BF/BW ratios.

### Histopathological studies

The BFs from all 65 animals were fixed in phosphate buffer containing 10% (v/v) formalin, embedded in paraffin and sectioned. The sections were stained with hematoxylin and eosin. Bursa lesion scores were determined using a light microscope and comparison between groups [17] as previously described [34]: score 0, no bursal damage or lesion in any follicle, clear demarcation of medulla and cortex; score 1, mild necrosis and mild lymphocyte depletion in a few follicles with overall bursal architecture maintained; score 2, moderate atrophy or lymphocyte depletion in one third to one half of the follicles, aggregation of heterophils, macrophages, and hyperplasia of epithelial reticular cells with some vacuole-like structures; score 3, more than half of the follicles show severe necrosis, atrophy and lymphocyte depletion, and loss of the outline of follicular architecture such that it is replaced with proliferating connective tissue and fibroplasias.

### Virus clearance assay

The presence of the IBDV antigen in the BF was analyzed by sandwich ELISA as previously described [24] with some modifications. Briefly, the wells of a flat-bottom 96-well microtiter plate were coated overnight at 4°C with 10 µg/ml mouse anti-IBD-VP2 antibody (Ingenasa, Madrid, Spain). The wells were washed with PBST, blocked by adding 150 µl/well 2.5% (w/v) skimmed milk in PBST and incubated for 1 h at room temperature. The BF was ground and diluted 1∶1 (w/v) in PBS and 100 µl of the homogenate was added to the wells. The plate was incubated overnight at 4°C before adding 100 µl rabbit anti-IBD-VP2 (diluted 1∶10,000 in PBST) and incubating for 1 h at room temperature. After washing again, we added 100 µl horseradish peroxidase-conjugated goat anti-rabbit secondary antibody diluted to 0.12 µg/ml (Dianova, Hamburg, Germany). The bound antibodies were detected using ABTS substrate and the absorbance was read at 405 nm on a microplate reader. Values greater than 0.2 were considered positive.

### Statistical analysis

Statistical analysis was carried out using SPSS v15 software (SPSS Inc., Chicago, IL, USA). Each chicken was considered as an experimental unit. The significance level (α) was set to 0.05. The normality of the data and the homogeneity of variances were assessed using Shapiro-Wilk's and Levene's tests, respectively. The collected data were subjected to one-way analysis of variance (ANOVA) followed by Duncan's test at significance level of p<0.05.

## Results

### Production of IBD-VP2 in Pichia pastoris

**Figure 2:**
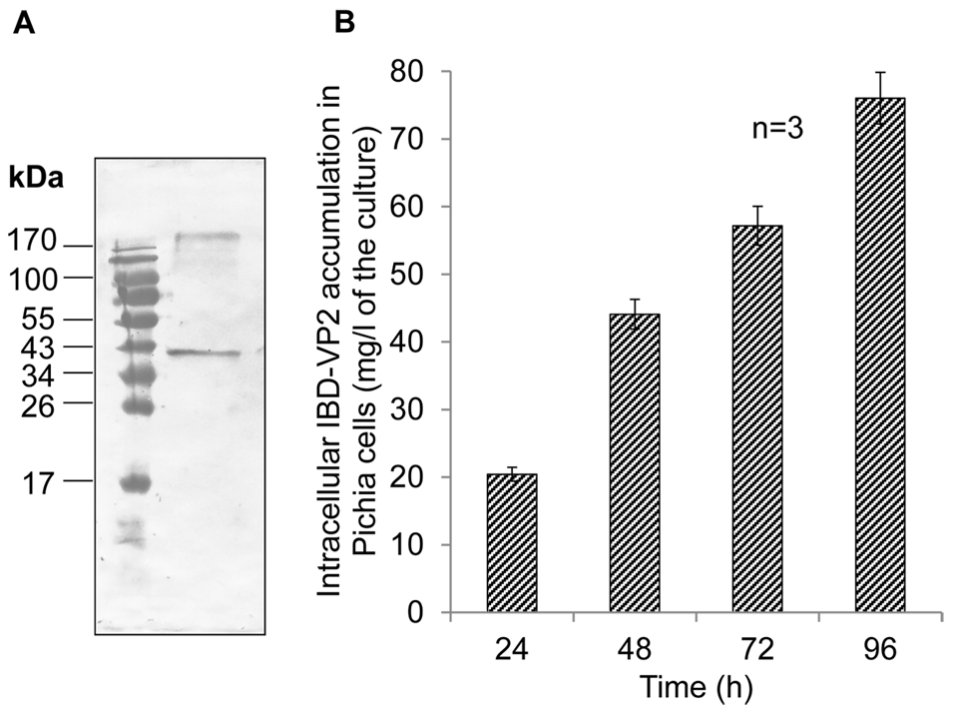
Characterization of recombinant IBD-VP2 produced in *Pichia pastoris*. (A) A 12-µl aliquot of the *P. pastoris* cell lysate was separated by SDS-PAGE (12% (w/v) polyacrylamide) and blotted onto a nitrocellulose membrane. The antigen was detected using a rabbit anti-IBD-VP2 (1∶10,000) primary antibody and an alkaline phosphatase-conjugated goat anti-rabbit secondary antibody (0.12 µg/ml). The signal was detected with NBT/BCIP for 5 min at room temperature. (B) Intracellular accumulation level of IBD-VP2 in *P. pastoris* cultures following expression induction. Biological triplicates were used for each experiment.

The 1323-bp mature *IBD-VP2* cDNA sequence was amplified from IBDV strain IR01 and transferred to the pPICZ_B vector, containing a C-terminal His_6_-tag sequence for protein detection and purification. The vector was linearized and introduced into *P. pastoris* cells, resulting in the insertion of the transgene at the *AOX1* locus. The recombinant yeast cells were then induced with methanol, and immunoblot analysis confirmed the presence of a 40-kDa VP2 band (Figure 2A) and a further band larger than 170 kDa that appeared because of the hydrophobic nature of the IBD-VP2 protein. The recombinant protein accumulated at levels of up to 76 mg/l of the culture after 96 h expression induction (Figure 2B).

### Purification and characterization of IBD-VP2

**Figure 3:**
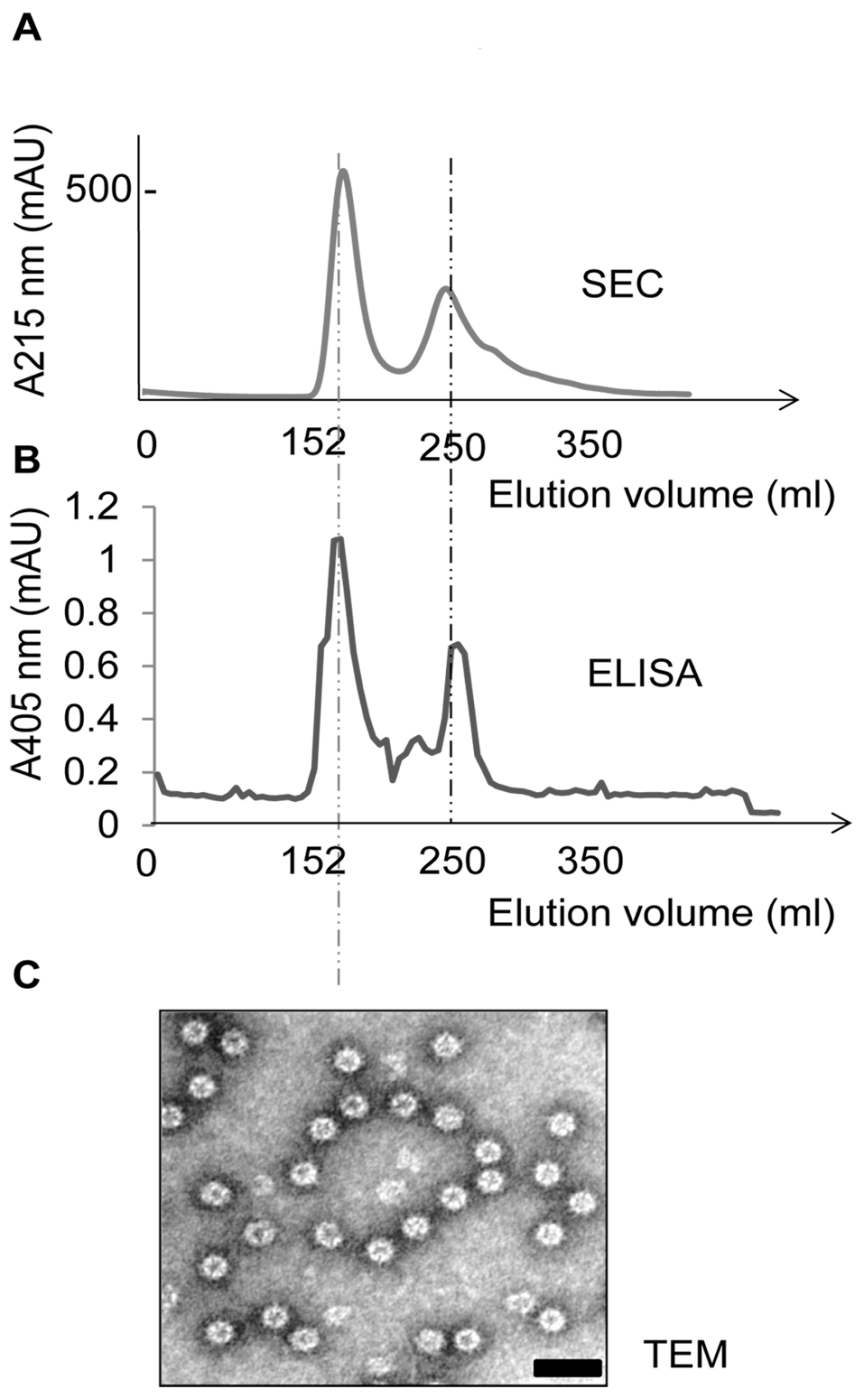
Separation of partially-purified yeast-derived IBD-VP2 by SEC and analysis by electron microscopy studies. (A) The IBD-VP2 protein was produced and extracted from the *P. pastoris* cells using breaking buffer (pH 4.0) followed by precipitation with 50% ammonium sulfate. The protein pellet was resuspended in PBS and separated by SEC using a S-400 HR column with a size separation range 20–8000 kDa. (B) The eluted fractions were tested for IBD-VP2 content using an indirect ELISA, revealing IBD-VP2 peaks at 152 and 250 ml during SEC. Electron microscopy revealed that only the 152-ml peak contained fully-assembled 23-nm IBD-SVPs. Scale bar = 50 nm.

An acidic extraction buffer (pH 4.0) allowed the recovery of 60% of the IBD-VP2 protein at a purity of 75%. Subsequent ammonium sulfate precipitation improved the purity of IBD-VP2 to 90% without substantial further loss. The remaining IBD-VP2 was purified further and simultaneously characterized by size exclusion chromatography (SEC). The eluent was collected in 2-ml fractions and a 10-µl sample from each fraction was analyzed by ELISA to determine the VP2 content. Two major peaks were detected by ELISA at 152 and 250 ml of the elution volume, both of which contained high and low molecular mass fractions of IBD-VP2 (Figure 3). Neither of these peaks was present in the wild-type Pichia X-33 cell extract purified under the same conditions (data not shown). Electron microscopy revealed that the 152-ml peak contained larger (23 nm) fully-assembled IBD-SVPs with a *T* = 1 lattice (Figure 3C) as previously described [35], [36], whereas symmetrical structures were not detected in the 250-ml peak. Fractions representing the 152-ml peak were collected, mixed and concentrated using nanosep centrifugal device with a 300-kDa molecular weight cut off (MWCO). As we previously reported [30], 38 mg of IBD-SVPs with >95% purity was extracted from the cells harvested from 1 l of culture using an acidic buffer, followed by ammonium sulfate precipitation and SEC.

**Figure 4:**
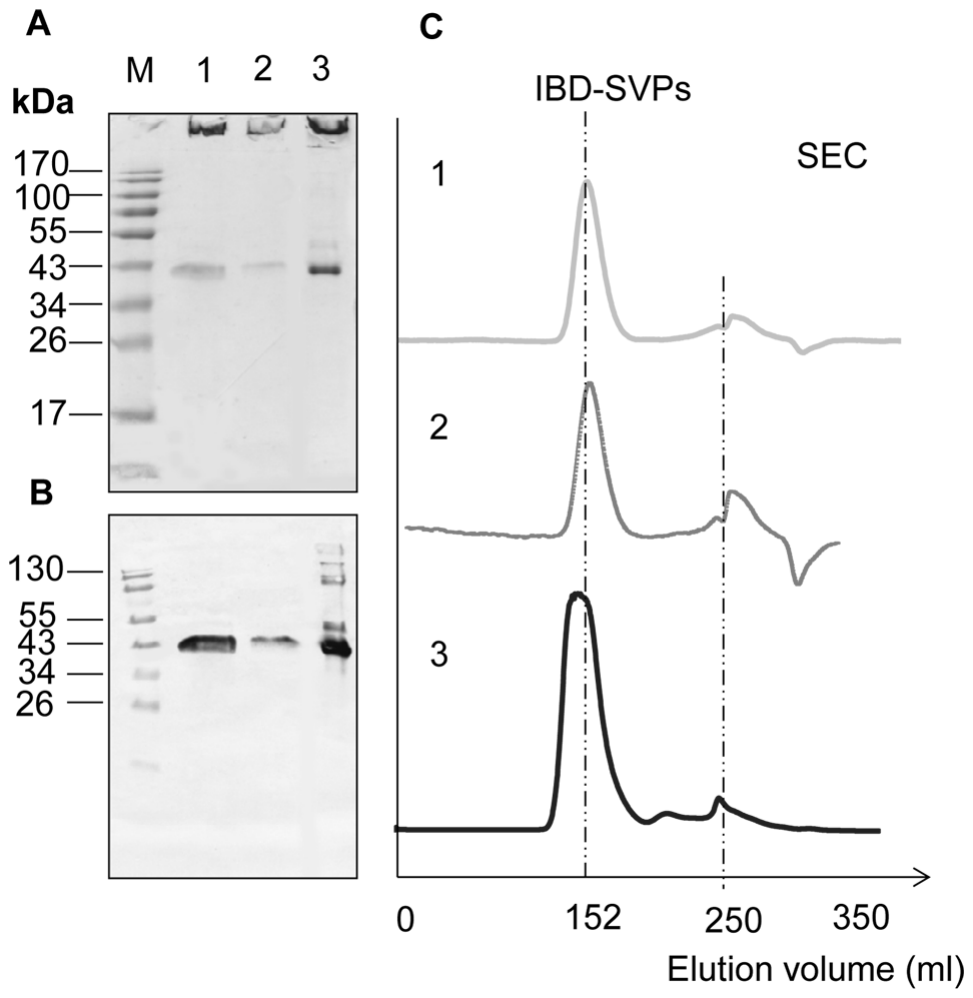
Analysis of IBD-SVP stability and integrity after yeast cell inactivation using SDS-PAGE and SEC. We separated 12-µl samples by SDS-PAGE (12% (v/w) polyacrylamide) followed by staining with Coomassie Brilliant Blue (A) and immunoblot detection (B) using a rabbit anti-VP2 primary antibody, an alkaline phosphatase-conjugated goat anti-rabbit secondary antibody (0.2 µg/ml) and NBT/BCIP to detect the signal: (1) purified IBD-SVPs derived from freeze-dried inactivated *P. pastoris* cells; (2) purified IBD-SVPs derived from active freeze-dried *P. pastoris* cells; (3) purified IBD-SVPs as a positive control. (C) Chromatogram obtained by separation of the corresponding samples using a SEC S-400 HR column and Äkta Explorer.

Recombinant yeast cells expressing IBD-VP2 were heat-inactivated, freeze-dried and ground to a fine powder as described above, and the IBD-SVPs were extracted and purified. Immunoblot analysis and SEC confirmed that IBD-VP2 remains stable during these treatments and the integrity of IBD-SVPs are maintained (Figure 4). We also calculated that 1 mg of freeze-dried yeast powder contains approximately 16 µg of VP2 (data not shown). The cell powder was stored at +4°C prior to immunization.

### Chicken immunization and serum antibody response

**Figure 5:**
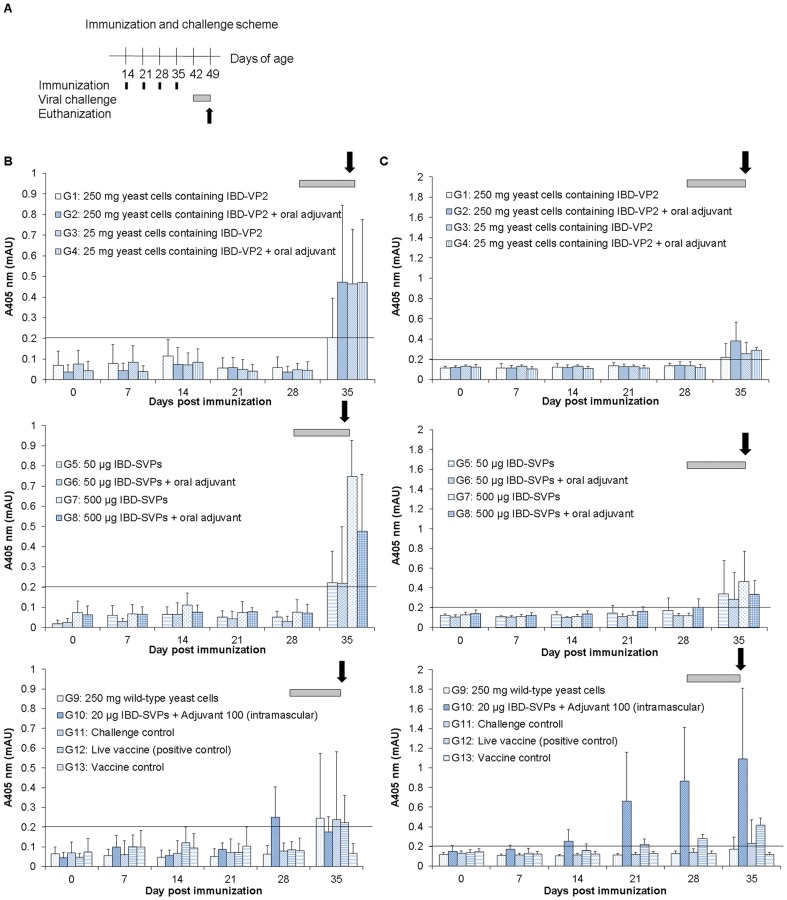
Immunization of chickens with IBD-VP2 or purified IBD-SVPs produced in *P. pastoris*. (A) Immunization scheme: two-week-old chickens were immunized at the indicated times (schematized as black bars) by either oral (groups 1–9 and 12) or intramuscular (group 10) administration. Oral immunization was carried out using different doses of either freeze-dried yeast (groups 1–4) or purified yeast-derived IBD-SVPs (groups 5–8) with and without an oral adjuvant mixture. Wild-type *P. pastoris* X-33 cells were used as a negative control (group 9) and Avipro Gumboro vac was used as a positive control (group 12). Intramascular immunization was carried out using 20 µg of purified yeast-derived IBD-SVPs mixed with Adjuvant 100 (Gerbu). Two groups remained unvaccinated, one in the challenge experiment and used as a challenge control (group 11) and the other unchallenged as a vaccine control (group 13). The gray bar indicates the time interval following viral challenge; arrows indicate the time of death. The reactivity of chicken sera IgM (B) and IgY (C) with IBD-SVPs was determined by ELISA. The serum samples were collected before immunization, before viral challenge and one week after challenge. The mean of absorbance at 405 nm is shown for five chickens in each group with standard error indicated by error bars. The 0.2 OD value was used as a cut off; chickens with a higher antibody titer were considered seropositive.

Two-week-old SPF chickens were immunized with purified IBD-SVPs or cell-encapsulated IBD-VP2 proteins. For each form of the antigen, two different doses were administered with and without adjuvant. The commercial attenuated IBDV vaccine Avipro Gumboro vac was used as a positive control, and inactivated freeze-dried wild-type *P. pastoris* cells were used as a negative control. The powdered cells were resuspended in 1 ml drinking water and the adjuvant, where required, was added immediately before administration. Two groups remained unvaccinated (groups 11 and 13) and one group received an intramuscular injection of the purified antigen (group 10). Vaccination was carried out weekly for 4 weeks followed by viral challenge (Figure 5A). The serum samples were collected before each immunization and challenge, and when the chickens were sacrificed for analysis.

Serum IgM and/or IgY induced by the vaccine and/or virus were detected by ELISA as shown in Figure 5B–C and Table 2. An IgM response before challenge was only detected in the chickens from group 10, which received the intramuscular vaccine, whereas this response appeared in all other chickens following viral challenge. In contrast, an IgY response was detected in groups 10 and 12 as well as groups that received 50 µg purified IBD-SVPs without adjuvant (group 5) and 500 µg purified IBD-SVPs with adjuvant (group 8) before challenge. The IgY response was comprehensive and potent in chickens from group 10 (intramuscular vaccine) and group 12 (oral attenuated virus).

**Table 2:** Analysis of the antibody response in immunized chickens before and after challenge with IBDV.

			IgM seropositive^2^		IgY seropositive		IDEXX ELISA seropositive		
Experimental groups	Administration route^1^	Vaccine composition	Before challenge	After challenge	Before challenge	After challenge	Before challenge	After challenge	Survivors^3^
1	O	250 mg of Pichia^4^ containing IBD-VP2	0/5	2/3	0/5	3/3	0/5	3/3	3/5
2	O	250 mg of Pichia containing IBD-VP2 + oral adjuvant^5^	0/5	5/5	0/5	5/5	0/5	5/5	5/5
3	O	25 mg of Pichia containing IBD-VP2	0/5	4/5	0/5	4/5	0/5	5/5	5/5
4	O	25 mg of Pichia containing IBD-VP2 + oral adjuvant	0/5	4/4	0/5	3/4	0/5	4/4	4/5
5	O	50 µg of purified IBD-SVPs	0/5	3/3	1/5	2/3	0/5	3/3	3/5
6	O	50 µg of purified IBD-SVPs + oral adjuvant	0/5	2/2	0/5	2/2	0/5	2/2	2/5
7	O	500 µg of purified IBD-SVPs	0/5	5/5	0/5	4/5	0/5	5/5	5/5
8	O	500 µg of purified IBD-SVPs + oral adjuvant	0/5	4/5	2/5	5/5	2/5	5/5	5/5
9	O	250 mg of Pichia wild-type	0/5	2/2	0/5	2/2	0/5	2/2	2/5
10	P	20 µg of purified IBD-SVPs + Adjuvant 100	3/5	2/5	5/5	5/5	3/5	5/5	5/5
11	X	Challenge control	0/5	2/2	0/5	2/2	0/5	2/2	2/5
12	O	AviPro Gumboro vac (oral control^6^)	0/5	3/5	5/5	5/5	5/5	5/5	5/5
13	X	Vaccine control	0/5	0/5	0/5	0/5	0/5	0/5	5/5

1 Route of vaccine administration was shown as O: oral (via oral gavage), P: parenteral (intramuscularly) and X: no vaccination.

2 Chickens with A405 nm above 0.2 were considered seropositive.

3 The ratio of the survivors to the total chickens in the group one week after viral challenge.

4 Pichia: freeze-dried and inactivated intact *P. pastoris* cells. Resuspended in 1 ml drinking water.

5 Oral adjuvant is a mixture of NaF salt (100 mg/kg chicken weight) and 50 µg CpG oligodeoxynucleotide (CpG ODN).

6 The administered dosage was according to the manufacture's instructions (Lohmann Animal Health, Cuxhaven, Germany).

Following virus challenge, chickens from groups 8, 10 and 12 as well as those that received 250 mg freeze-dried yeast cells containing IBD-VP2 with adjuvant (group 2) were protected from the virus and showed a positive IgY response. Although all chickens in the groups receiving 25 mg freeze-dried yeast cells containing IBD-VP2 (group 3) and 500 µg purified IBD-SVPs both without adjuvant (group 7) were fully protected, only 80% were IgY seropositive, and the remaining 20% were instead IgM seropositive. However, in other groups, some IgM seropositive but IgY seronegative chickens were not protected. Indeed, all surviving birds were seropositive in the IDEXX ELISA indicating that this method evaluates positive response resulting from either IgM or IgY.

### Health status and clinical signs

Weight gain during the experiment was monitored as a health index. No significant difference (p>0.05) in weight gain was observed among the groups during the immunization phase, which indicates that the vaccine did not interfere with normal growth (data not shown). Typical IBD clinical signs such as ruffled feathers, depression, abnormal feces and mortality were assessed daily following the challenge. Birds in the groups received intramascular vaccine (group 10) as well as those received live vaccine (group 12) showed no clinical sign following challenge. Clinical signs of IBD appeared 2 days post-infection (dpi) and declined by day 4 dpi due to the rapid recovery of survivors. At 2 dpi, moderate to severe clinical signs appeared among the mock-immunized birds (group 9) and challenge control (group 11). Most of the birds in the other groups developed mild clinical signs. At 3 dpi, mortality was observed among the mock-immunized birds (group 9) and challenge control (group 11) as well as birds in groups 5 and 6 that received 50 µg IBD-SVPs with and without oral adjuvant. The other chickens in these groups showed severe clinical signs of IBD. Other groups that were orally immunized with cell-encapsulated antigens showed mild to moderate clinical signs. Mortality was observed among these groups beginning 4 dpi but with a lower incidence (two in group 1 and one in group 4) than the mock-immunized and challenge control groups. Mild clinical signs also appeared by 4 dpi in birds from groups 7 and 8, which received 500 µg purified IBD-SVPs with and without oral adjuvant, but they recovered by 5 dpi. In contrast, mortality and moderate to severe clinical signs were observed in birds from groups 5 and 6, which received 50 µg IBD-SVPs. At 5 dpi, the survivors showed the first signs of recovery. These observations indicate that oral immunization with either cell-encapsulated or purified antigen is immunogenic but the potency of the resulting immunity is dose and formulation-dependent. In oral delivery, 500 µg of purified IBD-SVPs inhibits clinical signs more efficaciously than 50 µg of purified IBD-SVPs and even 250 mg of yeast cells containing IBD-VP2 (equivalent to 4 mg of the antigen).

### Efficacy of protection against viral challenge

Chickens from all groups (except group 13) were challenged with the classical IBDV strain MB3, and the post-challenge analysis is summarized in Table 3. The BF/BW ratios for the surviving chickens in groups 1–7 and those in the mock-vaccinated (group 9) and unvaccinated controls (group 11) differed significantly (p<0.05) from those in groups 8, 10, 12 and the unchallenged group 13. This indicated that, among the orally-vaccinated groups, only those receiving a high dose of the purified vaccine with adjuvant (group 8) were protected to the same degree as chickens administered with either attenuated virus or the parenteral vaccine.

**Table 3:** Summary of the results obtained from immunized chickens challenged with IBDV.

				Histopathological bursal lesion score^3^						
Experimental groups	Administration route^1^	Vaccine composition	BF/BW ratio^2^	0	1	2	3	Average	Antigen in bursa^4^	Survivors
1	O	250 mg of Pichia^5^ containing IBD-VP2	2.70 A	0	2	3	0	1.6	4/5	3/5
2	O	250 mg of Pichia containing IBD-VP2 + oral adjuvant^6^	2.24 A	0	5	0	0	1	5/5	5/5
3	O	25 mg of Pichia containing IBD-VP2	1.93 A	0	3	2	0	1.4	5/5	5/5
4	O	25 mg of Pichia containing IBD-VP2 + oral adjuvant	2.00 A	0	4	1	0	1.2	4/5	4/5
5	O	50 µg of purified IBD-SVPs	2.16 A	1	0	1	3	2.2	4/5	3/5
6	O	50 µg of purified IBD-SVPs + oral adjuvant	1.33 A	0	1	2	2	2.2	5/5	2/5
7	O	500 µg of purified IBD-SVPs	2.01 A	0	3	2	0	1.4	5/5	5/5
8	O	500 µg of purified IBD-SVPs + oral adjuvant	3.07 B	2	2	0	1	1	2/5	5/5
9	O	250 mg of Pichia wild-type	1.71 A	0	1	2	2	2.2	4/5	2/5
10	P	20 µg of purified IBD-SVPs + Adjuvant 100	3.72 B	5	0	0	0	0	0/5	5/5
11	X	Challenge control	1.48 A	0	2	0	3	2.2	5/5	2/5
12	O	AviPro Gumboro vac (oral control^7^)	3.16 B	5	0	0	0	0	0/5	5/5
13	X	Vaccine control	4.63 B	5	0	0	0	0	0/5	5/5

1 Route of vaccine administration was shown as O: oral (via oral gavage), P: parenteral (intramuscularly) and X: no vaccination.

2 BF/BW ratio was calculated by the bursal weight (g) divided by body weight (kg) and presented as the mean values that were presented by different letters (A and B) are significantly different (p<0.05).

3 The bursa lesion score was calculated according to previously described system [33].

4 Chickens with antigen were detected by sandwich ELISA.

5 Pichia: freeze-dried and inactivated intact *P. pastoris* cells. Resuspended in 1 ml drinking water.

6 Oral adjuvant is a mixture of NaF salt (100 mg/kg chicken weight) and 50 µg CpG oligodeoxynucleotide (CpG ODN).

7 The administered dosage was according to the manufacture's instructions (Lohmann Animal Health, Cuxhaven, Germany).

Bursa lesion scoring was carried out by comparing the histopathology of BFs isolated from chickens that had died during the experiment or that were killed at the end-point. The results indicated that chickens vaccinated with a high dose of the purified (group 8) or cell-encapsulated antigen (group 2) along with the adjuvant, showed less bursal damage than the other groups of orally-vaccinated chickens. Oral vaccination with either purified IBD-SVPs or cell-encapsulated IBD-VP2 prevented virus replication *in vivo* with varying efficacy. We found that 500 µg of the purified vaccine with adjuvant (group 8) achieved 60% viral clearance whereas the parenteral and oral control vaccines achieved 100% viral clearance.

The protection rate was 100% in the orally-vaccinated chickens from groups 2, 3, 7 and 8, and also in those receiving the intramuscular vaccine (group 10) and the live vaccine (group 12). This indicated that the oral administration of purified and cell-encapsulated IBD-SVPs is protective, although it does not eliminate the virus as efficiently as the live vaccine or parenteral IBD-SVPs.

## Discussion

Vaccination is currently the most effective way to control IBDV, and conventional vaccines are attenuated viruses based on mild or intermediate strains [37] thus avoiding vaccination side effects such as immunosuppression and clinical disease signs [1]. However, these vaccines are less effective against the diverse and vv IBDV strains that have arisen more recently. Recombinant subunit vaccines comprising the host-protective antigen (VP2) from those strains could provide a suitable alternative, and the lack of proliferation after vaccination would avoid the side effects attributed to the live vaccine. A rapid and reliable large-scale production platform is required to meet demand for vaccines against circulating IBDV strains, and the yeast *P. pastoris* is a simple and cost-effective eukaryotic platform for the production of heterologous proteins which meets these criteria. Transgene integration into the yeast genome allows the stable propagation of recombinant host cells, allowing us to achieve yields of 76 mg VP2 per liter of culture (16 mg/g of freeze-dried cells). Although VP2 as a protective recombinant vaccine has already been produced in other heterologous expression systems such as plants [16], [29], yeast [25], [38] and insect cell lines [26], [27], [28] with various degree of success, according to our knowledge the maximum reported production level was 30 mg/l in High-Five cells [27].

The recombinant IBD-VP2 protein was designed to accumulate in the cytosol, thus (i) preventing glycosylation in the secretory pathway (which could induce a non-favorable immune response), and (ii) encapsulating the antigen within the cell to provide protection against the harsh acidic and proteolytic conditions in the digestive tract following oral delivery. SDS-PAGE and immunoblot analysis of protein extracts from the recombinant yeast cells revealed the presence of a 40-kDa protein that was detected with a VP2-specific antibody, and further analysis showed that the IBD-VP2 subunits were aggregated into more immunogenic IBD-SVPs (Figure 3) that remains stable during heat inactivation and freeze drying (Figure 4).

We found that *P. pastoris* provided a practical system for antigen production and oral delivery to chickens. *P. pastoris* cells producing VP2 have previously been administered to chickens by intramuscular injection and were shown to be safe and to confer protection against IBDV [24]. We found that orally-administered heat-inactivated yeast cells producing IBD-VP2 induced a protective immune response against IBDV in chickens (Figure 5 and Table 2) which increased survival rates to 60–100% compared to 40% in the control groups. The oral delivery of 500 µg purified yeast-derived antigen conferred 100% protection, which is much better than the 10% protection previously achieved by oral immunization with heat-inactivated but intact cells of the yeast *Kluyveromyces lactis*, containing 1–3 mg IBD-VP2 [38]. In contrast, oral immunization with transgenic rice grains containing 2–10 mg VP2 conferred 83% protection [16]. The authors speculated that the dose of VP2 provided by *K. lactis* cells was insufficient to induce a potent immune response hence the low level of protection [38]. We found that 500 µg of purified yeast-derived IBD-SVPs was more immunogenic and protective than 4 mg of encapsulated VP2 and conferred full protection. We therefore concluded that homogenization of the cells may be essential to release the antigen and thus expose it to mucosal immunocompetent cells. It seems that the chicken digestive system does not digest the yeast cell walls rapidly enough to release the antigen. We also conclude that unencapsulated, purified IBD-SVPs are stable enough in the chicken digestive tract to resist degradation until they are taken by M cells, which allows them to induce the immunocompetent cells located beneath. This confirms our previous observation that IBD-SVPs tolerate acidic conditions (pH 2.0) *in vitro* without becoming denatured [30].

Although the oral delivery of VP2 was no better (in terms of protection, clinical signs, BF/BW ratios and virus clearance) than the current vaccination measures based on parenteral vaccination with IBD-SVPs and oral vaccination with live vaccine, the oral delivery of 500 µg purified IBD-SVPs conferred full protection and (when administered with adjuvant) achieved 60% viral clearance. This is promising because it suggests that oral adjuvants can induce a cytotoxic T-cell response. An optimized formulation and/or alternative adjuvants may improve the efficacy of the oral vaccine. Importantly, the oral delivery of subunit vaccines is quicker, safer and less expensive than parenteral vaccination because expert personnel are not required and the process does not cause stress in the chickens. In contrast to complete vaccines, recombinant subunit vaccines allow discrimination between infected and vaccinated animals (DIVA) based on the different antibody responses against the vaccine and the whole virus. Serological testing based on the detection of antibody responses against virus proteins that are not found in the vaccine will therefore facilitate the controlled eradication of the disease. Subunit vaccines can also be used without damaging the disease-free status of countries because discriminatory antibodies would not interfere with disease surveillance by serological testing [39].

Protection against IBDV is highly dependent on the antigenic correlation between the vaccine and the circulating virus strains [40]. Although all identified IBD-VP2 amino acid sequences are more than 93% identical, the variations between strains are generally found within virus-neutralizing epitopes. Vaccination with highly pathogenic strains confers protection against less pathogenic challenge strains. However, the dose and strain of both the vaccine and challenge virus affects the degree of protection [41], [42]. Cross-protection is useful for the development of vaccines conferring protection against a broad spectrum of IBDV strains, and we found that expressing IBD-VP2 derived from the vv strain IR01 as a recombinant protein in yeast results in the assembly of SVPs that protect chickens against the classical MB3 strain following oral vaccination. The serological cross reactivity among IBDV isolates may explain the observed protection. The vv strain IR01 with Asian origin is more that 99% identical at amino acid level to European strains. Therefore, the immunization and challenge experiments reflect the situation in the field. However, the immunological potency is dependent on dose and formulation, and could be thus improved by the development of optimized formulation strategies such as the homogenization of cell preparations and/or the co-administration of alternative adjuvants.
